# Genomic Analysis of Cardiovascular Diseases Utilizing Space Omics and Medical Atlas

**DOI:** 10.3390/genes16090996

**Published:** 2025-08-25

**Authors:** Ryung Lee, Abir Rayhun, Jang Keun Kim, Cem Meydan, Afshin Beheshti, Kyle Sporn, Rahul Kumar, Jacques Calixte, M. Windy McNerney, Jainam Shah, Ethan Waisberg, Joshua Ong, Christopher Mason

**Affiliations:** 1 Department of Medicine, Jacob’s School of Medicine and Biomedical Sciences, Buffalo, NY 14203, USA; abirrayh@buffalo.edu; 2 Department of Physiology, Biophysics and Systems Biology, Weill Cornell Medicine, New York, NY 10065, USA; jak4013@med.cornell.edu (J.K.K.); cem2009@med.cornell.edu (C.M.); chm2042@med.cornell.edu (C.M.); 3 Center for Space Biomedicine, McGowan Institute for Regenerative Medicine, Department of Surgery, University of Pittsburgh School of Medicine, Pittsburgh, PA 15261, USA; abehesht@broadinstitute.org; 4 Stanley Center for Psychiatric Research, Broad Institute of MIT and Harvard, Cambridge, MA 02142, USA; 5 Department of Medicine, Norton College of Medicine, 750 E Adams St., Syracuse, NY 13210, USA; spornk@upstate.edu; 6 Department of Medicine, University of Massachusetts Chan School of Medicine, Worcester, MA 01655, USA; rahulca13@gmail.com; 7 Vagelos College of Physicians and Surgeons, Columbia University, New York, NY 10032, USA; jc5968@cumc.columbia.edu; 8 Department of Psychiatry and Behavioral Sciences, Stanford University School of Medicine, Stanford, CA 94305, USA; windymc@stanford.edu; 9 Department of Veterans Affairs, VA Palo Alto Health Care System, Palo Alto, CA 94304, USA; 10 Department of Medicine, Albert Einstein College of Medicine, Bronx, NY 10461, USA; jainam.shah@einsteinmed.edu; 11 Department of Clinical Neurosciences, University of Cambridge, Cambridge CB2 0QQ, UK; ethanwaisberg@gmail.com; 12 Department of Ophthalmology and Visual Sciences, University of Michigan Kellogg Eye Center, Ann Arbor, MI 48105, USA; ongjo@med.umich.edu

**Keywords:** spaceflight, cardiovascular genomics, atherosclerosis, arrhythmia, immune pathways, astronaut health

## Abstract

Background: The Space Omics and Medical Atlas (SOMA) is an extensive database containing gene expression information from samples collected during the short-duration Inspiration4 spaceflight mission in 2021. Given our prior understanding of the genetic basis for cardiovascular diseases in spaceflight, including orthostatic intolerance and cardiac deconditioning, we aimed to characterize changes in differential gene expression among astronauts using SOMA-derived data and curated cardiovascular pathways. Methods: Using the KEGG 2021 database, we curated a list of genes related to cardiovascular adaptations in spaceflight, focusing on pathways such as fluid shear stress and atherosclerosis, lipid metabolism, arrhythmogenic ventricular hypertrophy, and cardiac muscle contraction. Genes were cross-matched to spaceflight-relevant datasets from the Open Science Data Repository (OSDR). Differential expression analysis was performed using DESeq2 (v1.40.2, R) with normalization by median-of-ratios, paired pre-/post-flight covariates, and log2 fold change shrinkage using apeglm. Differentially expressed genes (DEGs) were defined as |log2FC| ≥ 1 and FDR < 0.05 (Benjamini–Hochberg correction). Module score analyses were conducted across SOMA cell types to confirm conserved cardiac adaptation genes. Results: A total of 185 spaceflight-relevant genes were analyzed. Statistically significant changes were observed in immune-related cardiovascular pathways, particularly within monocytes and T cells. Persistent upregulation of arrhythmogenic genes such as GJA1 was noted at post-flight day 82. WikiPathways enrichment revealed additional pathways, including focal adhesion, insulin signaling, and heart development. Conclusions: Short-duration spaceflight induces significant gene expression changes that are relevant to cardiovascular disease risk. These changes are mediated largely through immune signaling and transcriptional regulation in peripheral blood mononuclear cells. Findings highlight the need for tailored countermeasures and longitudinal monitoring in future long-duration missions.

## 1. Introduction

Changes in human physiology occur at the molecular level in the context of spaceflight. This was demonstrated early in the NASA Twin Studies, where astronaut Scott Kelly experienced telomere elongation, genome instability, transcriptional/metabolic changes, and DNA methylation in immune and oxidative stress-related pathways [1,2]. The SpaceX 2021 Inspiration4 mission, which collected samples to build a sizeable multiomics database, has replicated some of the findings from the NASA Twin Study, including the lengthening of telomeres during spaceflight and changes in differential gene expression [3,4]. The five hazards of spaceflight are the main driving forces [5].

This hazardous spaceflight environment presents additional risk factors for the formation of cardiovascular diseases, including cardiac deconditioning. Cardiac deconditioning is more likely to arise during long-duration spaceflight [6]. While common, the mechanisms of action have not yet been elucidated. During short-duration spaceflight missions, like the Apollo missions, astronauts used 24 h Holter monitors in both in-flight and extravehicular activities (EVAs), exhibiting premature ventricular contractions during EVAs and transitions, along with additional supraventricular abnormalities [6]. Like cardiac deconditioning, the mechanisms of the cardiac dysrhythmia were also not yet made clear, although rises in catecholamines were suspected [7]. In follow-up monitoring through the Longitudinal Study of Astronaut Health (LSAH), cardiac arrhythmias were found to develop at an earlier age, despite selection for overall fitness, compared to the general population [8]. Furthermore, researchers have demonstrated left ventricular atrophy and left atrial hypertrophy when comparing cardiac structures using Magnetic Resonance Imaging (MRI) during the pre-flight and post-flight periods [8,9]. The structural changes may further exacerbate electrophysiological conduction, necessitating a deeper, more comprehensive understanding of the pathophysiological mechanisms underlying cardiovascular disturbances before exploration class missions. Thus, on exploration class missions, where greater exposure to space radiation arises, radiation-induced atherosclerosis is another high-priority concern [10].

**Figure 1:**
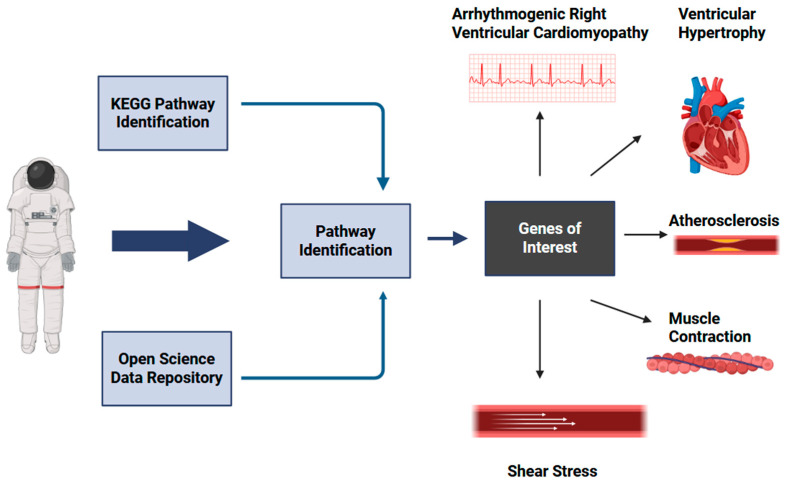
Proposed molecular model for spaceflight’s effects on cardiovascular pathology development in spaceflight. Significant presentations include fluid shear stress, arrhythmogenic right ventricular cardiomyopathy, atherosclerosis, muscle contraction, and hypertrophic cardiomyopathy. Created in BioRender. Lee, R. (2025) https://BioRender.com/bfzvxl0**.** URL accessed on 21 August 2025.

In this study, we utilized the publicly available genetic databases including the Open Science Data Repository (OSDR), the Kyoto Encyclopedia Genes and Genomes (KEGG), and the Space Omics and Medical Atlas (SOMA) to investigate whether known molecular interaction, reaction, and relation networks of cardiovascular diseases, including fluid shear stress, lipid, hypertrophic cardiomyopathy, arrhythmogenic right ventricular cardiomyopathy, and cardiac contraction (Figure 1), have changed differential expression in spaceflight [3,11]. The SOMA is the most prominent molecular atlas to date of the effects of spaceflight on the human body, comprising nearly 3000 samples from SpaceX’s 2021 Inspiration4, the NASA Twin Study, and JAXA missions. We begin with a system-level perspective using KEGG pathway enrichment and progressively narrow our focus to gene-level signatures derived from the SOMA dataset. We aim to uncover molecular mechanisms underlying structural cardiac changes and the heightened risk of cardiovascular disease observed during and after spaceflight (Figure 1).

## 2. Methods

To select our genes of interest, we utilized the Kyoto Encyclopedia Genes and Genomes (KEGG) PATHWAY database, a resource for understanding high-level functions and utilities of biological systems from molecular-level information. Using the cardiovascular system as the target system of interest, we selected our categories of interest: the circulatory system and cardiovascular disease. Given our prior understanding of cardiac physiology in spaceflight, we selected cardiac muscle contraction within organismal systems. Similarly, within human diseases we selected the following: lipids and atherosclerosis, fluids shear stress, hypertrophic cardiomyopathy, and arrhythmogenic right ventricular cardiomyopathy. Given that KEGG pathways are derived from terrestrially bound genomic systems, we cross matched our reference KEGG genome to OSDR studies that are relevant to our system of interest, which generated a truncated gene list of the same pathways in the context of spaceflight. Gene lists included the following hits from OSDR studies:

- Transcriptomic Effects on the Mouse Heart Following 30 Days on the ISS;
- RRRM-2 Mouse Heart Transcriptional Profiling;
- RR-3 Bulk and Spatial Heart Transcriptomics;
- JAXA Cell-Free RNA Analysis (Astronaut Plasma);
- Spaceflight Modulates Gene Expression in Astronauts;

We then performed a module score analysis of our genes against SOMA to examine score changes over the course of spaceflight (pre-flight, 1 day, 45 days, and 82 days post-flight). The SOMA does not include myocardial tissue; thus, we cross referenced KEGG cardiovascular disease pathways with OSDR datasets. Gene expression counts from the SOMA were analyzed using DESeq2 (v1.40.2, R). Data were normalized by the median-of-ratios method. Paired designs were incorporated for pre- and post-flight comparisons. Log2 fold changes were estimated with apeglm shrinkage. Genes with |log2FC| ≥ 1 and adjusted *p* < 0.05 (FDR) were considered significant. Differentially expressed genes (DEGs) were initially identified at a nominal threshold of *p* < 0.05. The statistical values selected for this dataset are described elsewhere [3]. We recognize the challenges of multiple-comparison adjustment in small astronaut cohorts, where formal false discovery rate (FDR) correction often results in uniform non-significance. Therefore, our analysis emphasizes genes that demonstrate consistent expression changes across multiple immune subtypes and timepoints, and that align with previously reported spaceflight-associated gene signatures. This approach provides biological robustness despite the exploratory statistical framework.

The cell lineages examined were derived from peripheral blood mononuclear cells including myeloid and lymphocytic derived cells. Additionally, pathway analysis was performed using the MSigDB database, WikiPathways collection, for broad capturing of various related pathways. Normalized Enrichment Score (NES) and *p*-values were calculated and deemed significant if they were less than our alpha value of 0.05.

## 3. Results

### 3.1. Genomics of Spaceflight Cardiovascular Risk

**Figure 2:**
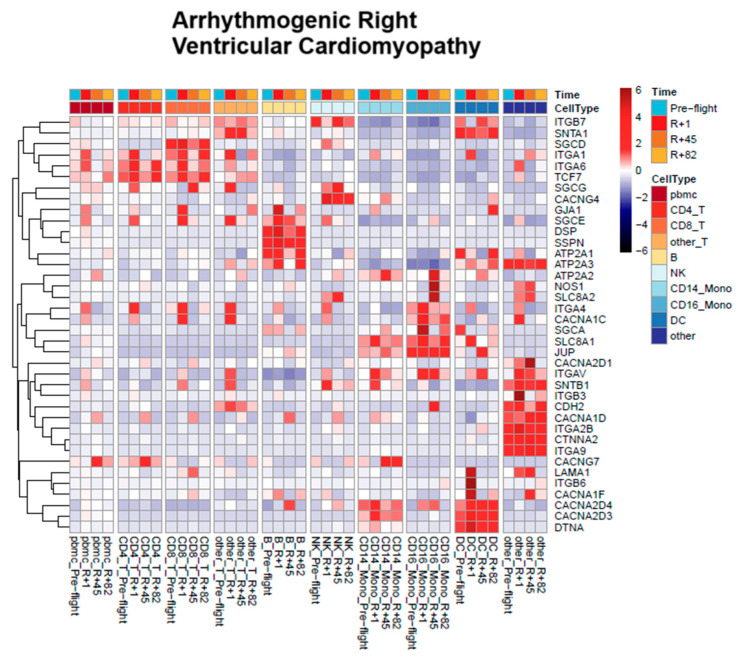
Heatmap displaying scaled log2 FC values for genes involved in arrhythmogenic pathways across immune cell types and spaceflight phases: pre-flight, post-flight (R) [R + 1, R + 45 and R + 82]. Rows represent individual genes and columns represent single-cell transcriptomes grouped by cell type (color-coded, e.g., CD4+ T, CD8+ T, NK, Monocytes, DCs) and spaceflight phase (top bar). Hierarchical clustering was applied to both genes and samples. Red indicates regulation, and blue indicates downregulation relative to OSDR cohort mean. Unless otherwise specified, gene-level results reflect FDR-adjusted tests (padj), and pathway/cytokine results report q-values (FDR) that are consistent with SOMA reporting.

We analyzed differential gene expressions using the publicly available SOMA database to identify molecular drivers of cardiovascular risk in spaceflight. Our analysis results are summarized in a heat map of the log2fold expression of candidate genes associated with cardiovascular conditions commonly impacted by microgravity and spaceflight for our selected KEGG pathways (Figure 2 and Supplementary Figure S1). WikiPathways provides additional extended pathways that are relevant to spaceflight from known cardiovascular systems.

### 3.2. SOMA Module Score Analysis

A total of 185 genes relevant to spaceflight were analyzed within the SOMA database. Across the cell lineages, various genes within each KEGG pathway demonstrated significant log2fold changes. These changes occurred on post-flight day 1. Overall, the strongest amount of change was upregulation with 6-fold changes in gene expression occurring within some immune cell populations. Many of the pathway genes recovered by post-flight period 82; however, some genes, such as GJA1 in the arrhythmogenic right ventricular cardiomyopathy pathways, did not recover after spaceflight in dendritic and B cells. From the analyzed immune cell types, T cells and peripheral mononuclear blood cells demonstrated the greatest amount of gene regulation. Changes in gene expression were conserved across immune cell types. Genes with significant regulation are summarized in Table 1.

### 3.3. WikiPathways

**Figure 3:**
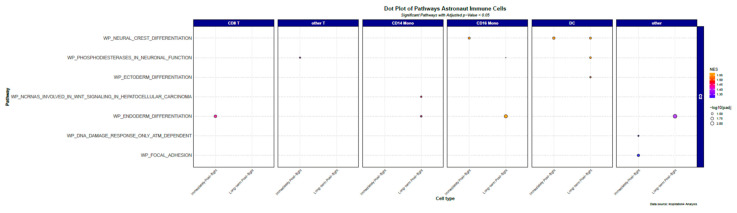
Dot plot of astronaut immune cells generated by comparing cardiovascular disease gene clusters to MSigDB WikiPathways categories using hypergeometric survival function. Values were filtered for padj < 0.05. Bubble color reflects Normalized Enrichment Score (NES) and size indicates statistical significance as –log_10_(adjusted *p*-value).

Following cross-matching of our gene list into the WikiPathways database, we identified several novel pathways implicated in spaceflight. Pathways with potential relevance to cardiovascular physiology included focal adhesion, heart development, insulin signaling, and nonalcoholic fatty liver disease, among others. In addition, the enrichment of pathways associated with cellular stress response, metabolism, and extracellular matrix remodeling was observed. These findings suggest systemic influences of spaceflight on signaling networks beyond the immune system, with implications for cardiovascular and metabolic health. Importantly, pathway enrichment patterns were largely conserved across immune cell types, consistent with PBMCs acting as systemic sensors of physiological adaptation. Figure 3 and Supplementary Figure S2 illustrate the spectrum of upregulated pathways derived from WikiPathways analysis.

## 4. Discussion

Rather than the direct involvement of the cells of the myocardium, these transcriptional shifts are occurring at the systemic level, reflected in PBMCs and plasma biomarkers. They do not imply that PBMCs are themselves mediating myocardial remodeling, but rather that circulating immune and plasma signatures provide accessible surrogates of cardiovascular adaptation. Importantly, the SOMA does not include myocardial tissue; instead, it profiles astronaut PBMCs at a single-cell resolution and a wide array of plasma-based omics (proteomics, cfRNA, cfDNA, metabolites). As such, our study evaluates systemic and immune-mediated surrogates of cardiovascular adaptation, rather than cardiomyocyte expression per se. This is particularly relevant for processes such as atherosclerosis, which is primarily immune-driven, and for arrhythmogenic pathways that may be reflected in circulating immune and plasma signals. The genes can be further categorized into their main immune function which includes innate immunity (*ACE, AKT3, APOB, ATF4, BCL2L1, CAMK2A, CAMK2B, CCL2, CD36, CTSL, CXCL1, CYBB*), adaptive immunity (*ASPH, CACNG4, CACNG7, CCL5, GJA1, ITGB3, KDR, TGFB3*), cytokine signaling (*AGER, ATF6, CACNA1C, EIF2AK3, EIF2S1, GSK3B, HSPA1A, HSPA1B, HSPA2, HSPA8*), apoptosis regulation (*ACTC1, CASP3, CASP8),* and *oxidative stress (ATP2A1, CDH2, COX1, COX5A, COX5B, COX6A3, COX6B1, COX6B2, COX6C, GSTA2*). This cardiovascular-focused analysis confirms the changes demonstrated in previous studies on longer-duration spaceflight [3]. The findings from this study corroborate previous cardiovascular DEG directionality. For instance, the *ITGA4* gene is known to be involved with endocardial differentiation and cardiac morphogenesis. *ITGA4* is also found on tissue resident memory T cells, stabilizing their retention and mobility in tissues [12]. The upregulation of *ITGA4* may be an inflammatory response, promoting smooth muscle cell transition to myofibroblasts and their proliferation. Still, other genes, such as *ATP1A3*, are crucial in maintaining the electrical environment of cardiomyocytes and may correspond with the changes in rhythm seen in spaceflight [13]. We adopted SOMA-consistent thresholds (|log2FC| > 0.5; FDR < 0.05) to facilitate comparability with the SOMA resource and prior astronaut omics studies, while acknowledging that small astronaut cohorts limit power; therefore findings are interpreted in a hypothesis-generating framework. We discuss the role of the immune cells in the development of cardiovascular disease below.

### 4.1. Molecular Basis of Disease

The immune system plays a pivotal role in the pathogenesis and progression of cardiovascular diseases (CVDs) through a dynamic interplay between innate and adaptive immune cells, cytokine signaling, and tissue remodeling mediators. Chronic inflammation has emerged as a central driver of atherosclerosis, myocardial infarction, heart failure, and cardiomyopathy, with molecular pathways linking immune cell activation to the structural and electrophysiological remodeling of cardiac tissue.

Monocytes and macrophages dominate the initial inflammatory response in CVD, orchestrated through chemokine gradients such as the CCL2-CCR2 axis [14]. CCL2, secreted by endothelial and smooth muscle cells in response to IL-1, IL-6, and TNF-α, promotes monocyte recruitment to sites of vascular injury and atherosclerotic plaque formation [14]. These cytokines also directly modulate cardiomyocyte ion channels, contributing to arrhythmogenesis by decreasing potassium currents and increasing calcium influx [15].

Within the adaptive immune compartment, CD4^+^ T cells, including T-helper and regulatory T cells (Tregs), are key modulators of vascular and myocardial inflammation. Tregs exert cardioprotective effects via anti-inflammatory cytokines such as IL-10 and TGF-β [16]. Their deficiency has been associated with enhanced lesion progression in atherosclerosis, abdominal aortic aneurysm (AAA), and post-MI remodeling [16]. In murine AAA models, Treg supplementation dose-dependently suppressed MMP-2 and MMP-9 expression, attenuating vascular degradation [16]. Conversely, excessive TNF-α production by effector T cells induces dilated cardiomyopathy through inflammatory infiltration and adverse ventricular remodeling.

Matrix metalloproteinase-9 (MMP-9), secreted by activated neutrophils, macrophages, and lymphocytes, serves as both a marker and a mediator of cardiac remodeling. MMP-9 facilitates extracellular matrix degradation and promotes inflammatory signaling via NF-κB and AP-1 activation [17]. Elevated MMP-9 levels correlate with adverse left ventricular (LV) remodeling, reduced ejection fraction, and persistent inflammation in chronic heart failure [17].

Transcription factor 7 (TCF7), highly expressed in plaque-resident T cells, contributes to immune-mediated vascular pathology by regulating the NF-κB/AKT/STAT1 axis. These transcriptional programs facilitate macrophage recruitment and cytokine production in atherosclerosis and may influence arrhythmogenic remodeling in diseases such as arrhythmogenic right ventricular cardiomyopathy [18]. In parallel, TGF-β signaling exerts nuanced effects, simultaneously suppressing macrophage activation while modulating fibroblast gene expression and lymphocyte function, thus acting as a mediator of both resolution and fibrosis depending on the context [19]. Together, these immune-driven molecular mechanisms underscore the dual role of inflammation in cardiovascular disease as both a protective and pathological force, and highlight potential therapeutic targets ranging from cytokine signaling to regulatory immune cell pathways.

### 4.2. Countermeasures

Given the increased risk of cardiovascular diseases on spaceflight missions, we discuss the currently available countermeasures and their relevance to cardiovascular diseases. Inflight exercises are known to reduce the progression of muscle atrophy in the spaceflight environment. Current equipment includes a treadmill with vibration isolation, stabilization, and advanced resistance exercise devices [20]. Standard and high-resistance exercises have ben demonstrated to prevent up to a 6% VO2 max and are efficacious against cardiac functionality, ventricular mass changes, and arrhythmias. Potential countermeasures for cardiac disturbances resulting from differential gene expressions in spaceflight may be tailored to the underlying disease etiology. Next, cardiovascular medications available on NASA medical kits include lisinopril, nitroglycerin, and metoprolol. Metoprolol is a selective beta-1 blocker, indicated for the treatment of angina, heart failure, hypertension, and arrhythmias, such as atrial fibrillation. Furthermore, in sudden cardiac death, nitroglycerin may serve as a temporizing measure for the development of ischemia. Lisinopril may also be beneficial, as it helps manage blood pressure and prevents further atrial hypertrophy [20].

However, current cardiovascular medications do not address the full spectrum of spaceflight-related cardiovascular disease [21]. Compression garments, including the 3-piece gradient compression garment, work by adding a force of 40–45 mmHg at the ankle, compressing the capacitance vessels [22]. It has been shown to prevent tachycardia and cause drops in stroke volume during orthostasis while maintaining systolic blood pressure. Similarly, cooling dissipates heat stress among astronauts, which is crucial during landing [23]. The additional lowering of temperature is thought to control tachycardia effectively for arterial pressures and further prevents structural changes and radiation-induced atherosclerosis. Atherosclerosis is mediated by endothelial dysfunction, oxidative stress, and inflammatory pathways; for atherosclerosis, both nutritional countermeasures and radiation shielding, including sulfhydryl or thiol antioxidants, may be useful. Gamma tocotrienol, an isoform of vitamin E, is known to mitigate the effects of oxygen ions and irradiation on cardiac function [24].

A key limitation is that our differential expression results were based on nominal *p*-values without formal multiple-comparison correction. Given the small number of astronauts, standard FDR procedures inflate to near 1.0, making interpretation impractical. We therefore present our findings as hypothesis-generating, with emphasis on biologically plausible changes conserved across immune subtypes and timepoints (e.g., *GJA1*, *ITGA4*). Importantly, these results are consistent with prior spaceflight transcriptomic studies, which strengthens confidence despite statistical constraints.

## 5. Conclusions

In summary, this study provides evidence for the increased risk of cardiovascular disease through differential gene expression in spaceflight. Our samples, primarily from a short-duration low Earth orbit mission, suggest that risks may escalate in longer missions, such as Mars exploration. Limitations of the current study include small sample sizes—there were only 14 crewmembers from which we derived the SOMA biospecimen samples. Furthermore, the 3-day mission duration limits findings to the acute spaceflight adaptation phase. However, as a commercial spaceflight mission, Inspiration4 highlights the need for further clinical studies and longitudinal data points.

## Figures and Tables

**Table 1:** Primary cardiovascular adaptation genes and their changes in spaceflight.

Cardiovascular Adaptation	Gene Name	KEGG Pathway	Change in Microgravity	Cell Type
Cardiac Hypertrophy	*ATP2A1*	Muscle Contraction	Upregulated	CD8_T, CD14_Mono, DC
Cardiac Hypertrophy	*SGCG*	Hypertrophic Cardiomyopathy	Upregulated	PBMC, CD4_T, CD8_T, NK
Cardiac Hypertrophy	*ITGA6*	Hypertrophic Cardiomyopathy	Upregulated	PBMC, CD4_T, CD8_T
Cardiac Hypertrophy	*ITGA1*	Hypertrophic Cardiomyopathy	Upregulated	PBMC, CD4_T, CD8_T, CD14_Mono, DC
Cardiac Hypertrophy	*ITGB7*	Hypertrophic Cardiomyopathy	Upregulated	PBMC, CD4_T
Cardiac Hypertrophy	*ATP2A1*	Hypertrophic Cardiomyopathy	Upregulated	CD8_T, CD14_Mono
Cardiac Hypertrophy	*SGCD*	Hypertrophic Cardiomyopathy	Upregulated	PBMC, CD4_T, CD8_T
Atherosclerosis	*AKT3*	Fluid Shear Stress	Upregulated	CD14_Mono, DC
Atherosclerosis	*MAPK13*	Fluid Shear Stress	Upregulated.	CD14_Mono, DC
Atherosclerosis	*HSP90AA1*	Fluid Shear Stress	Upregulated.	CD14_Mono, DC
Atherosclerosis	*CASP8*	Lipid and Atherosclerosis	Upregulated	CD8_T, CD14_Mono
Atherosclerosis	*CASP3*	Lipid and Atherosclerosis	Upregulated.	CD8_T, CD14_Mono, DC
Atherosclerosis	*HSPA1A*	Lipid and Atherosclerosis	Upregulated.	CD14_Mono, DC
Atherosclerosis	*HSP90AA1*	Lipid and Atherosclerosis	Upregulated.	CD14_Mono, DC
Cardiac Arrhythmia	*ITGB7*	Arrhythmogenic Right Ventricular Hypertrophy	Upregulated	PBMC, CD4_T
Cardiac Arrhythmia	*SNTG1*	Arrhythmogenic Right Ventricular Hypertrophy	Upregulated	PBMC, CD4_T
Cardiac Arrhythmia	*KCNQ1*	Arrhythmogenic Right Ventricular Hypertrophy	Upregulated	PBMC, CD4_T, CD8_T
Cardiac Arrhythmia	*SGCD*	Arrhythmogenic Right Ventricular Hypertrophy	Upregulated	PBMC, CD4_T, CD8_T, CD14_Mono, DC
Cardiac Arrhythmia	*ITGA1*	Arrhythmogenic Right Ventricular Hypertrophy	Upregulated	PBMC, CD4_T, CD8_T
Cardiac Arrhythmia	*ITGA6*	Arrhythmogenic Right Ventricular Hypertrophy	Upregulated	PBMC, CD4_T, CD8_T, NK
Cardiac Arrhythmia	*SGCG*	Arrhythmogenic Right Ventricular Hypertrophy	Upregulated	PBMC, CD4_T, CD8_T, B, CD14_Mono
Cardiac Arrhythmia	*GJA1*	Arrhythmogenic Right Ventricular Hypertrophy	Upregulated	CD8_T, CD14_Mono

## Supplementary Materials

The following supporting information can be downloaded at https://www.mdpi.com/article/10.3390/genes16090996/s1, Figure S1: Heatmaps for additional KEGG Pathways; Figure S2: WikiPathways.

## Abbreviations

The following abbreviations are used in this manuscript:

ACE	Angiotensin-Converting Enzyme
AAA	Abdominal Aortic Aneurysm
AP-1	Activator Protein 1
ATP2A1	Sarco/Endoplasmic Reticulum Calcium-ATPase 1
B	B Lymphocyte
CAMK2A/B	Calcium/Calmodulin-Dependent Protein Kinase II Alpha/Beta
CD4_T	CD4+ T Lymphocyte
CD8_T	CD8+ T Lymphocyte
CD14_Mono	CD14+ Monocyte
COX	Cytochrome c Oxidase
CVD	Cardiovascular Disease
DC	Dendritic Cell
DEG	Differentially Expressed Gene
EVA	Extravehicular Activity
GJA1	Gap Junction Protein Alpha 1
HRC	Histidine-Rich Calcium-Binding Protein
IL	Interleukin
JAXA	Japan Aerospace Exploration Agency
KEGG	Kyoto Encyclopedia of Genes and Genomes
LSAH	Longitudinal Study of Astronaut Health
MAPK	Mitogen-Activated Protein Kinase
MMP-2/9	Matrix Metalloproteinase-2/9
MRI	Magnetic Resonance Imaging
MSigDB	Molecular Signatures Database
MYH6/7	Myosin Heavy Chain 6/7
NES	Normalized Enrichment Score
NF-κB	Nuclear Factor kappa-light-chain-enhancer of activated B cells
NK	Natural Killer Cell
OSDR	Open Science Data Repository
PBMC	Peripheral Blood Mononuclear Cell
RRRM	Rodent Research Reference Mission
SOMA	Space Omics and Medical Atlas
SNTB1	Syntrophin Beta 1
SNTG1	Syntrophin Gamma 1
TCF7	Transcription Factor 7
TGF-β	Transforming Growth Factor Beta
TNF-α	Tumor Necrosis Factor Alpha
TP53	Tumor Protein p53
VO2 max	Maximal Oxygen Consumption
WP	Community-curated Biological Pathways Database
