# Supplementary material for: Genomic Analysis of Cardiovascular Diseases Utilizing Space Omics and Medical Atlas
Source: Genes (Basel). 2025 Aug 25;16(9):996. doi: 10.3390/genes16090996 (PMC12469784; doi:10.3390/genes16090996)
Supplement: Supplementary file 1 [file genes-16-00996-s001.zip › genes-3771036-supplementary.pdf]

## Supplementary File Index

**Figure S1:** Heatmaps for additional KEGG Pathways. Heatmap display scaled $\log_2$ FC values for genes involved in KEGG cardiovascular disease pathways across immune cell types and spaceflight phase: pre-flight, R+1, R+45 and R+82. Rows represent individual genes and columns represent single-cell transcriptomes grouped by cell type (color-coded: e.g., CD4+ T, CD8+ T, NK, Monocytes, DCs) and spaceflight phase (top bar). Hierarchical clustering was applied to both genes and samples. Red indicates regulation, blue indicates downregulated relative to OSDR cohort mean.

**Figure S2:** Heatmaps Dot plot of astronaut immune cells generated by comparing cardiovascular disease gene clusters to MSigDB WikiPathways categories using hypergeometric survival function. Values were filtered for padj $< 0.05$. Bubble color reflects Normalized Enrichment Score (NES) and size indicates statistical significance as $-\log_{10}$(adjusted p-value).

**Figure S1:** Heatmaps for additional KEGG Pathways.

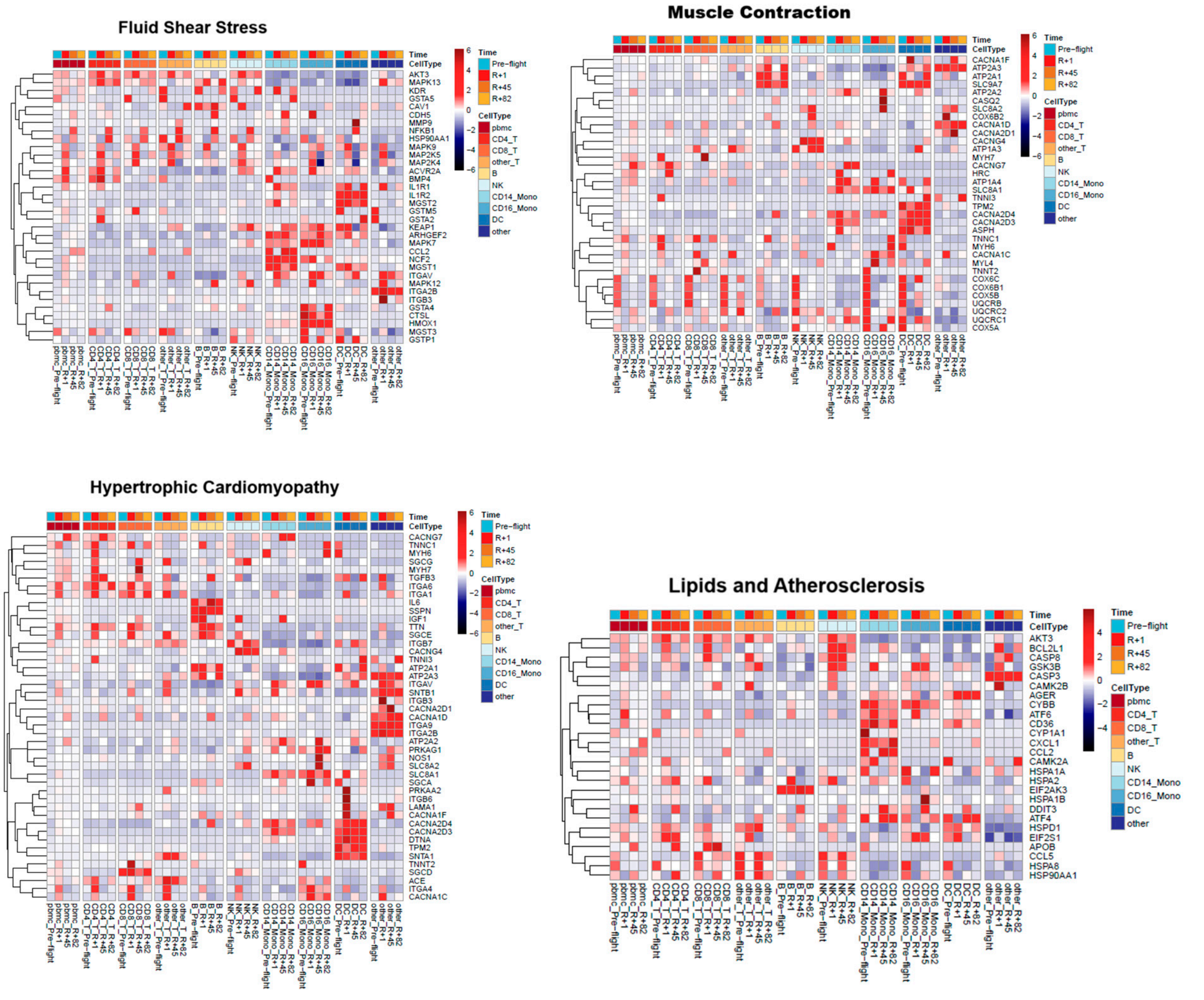

**Figure S2:** WikiPathways.

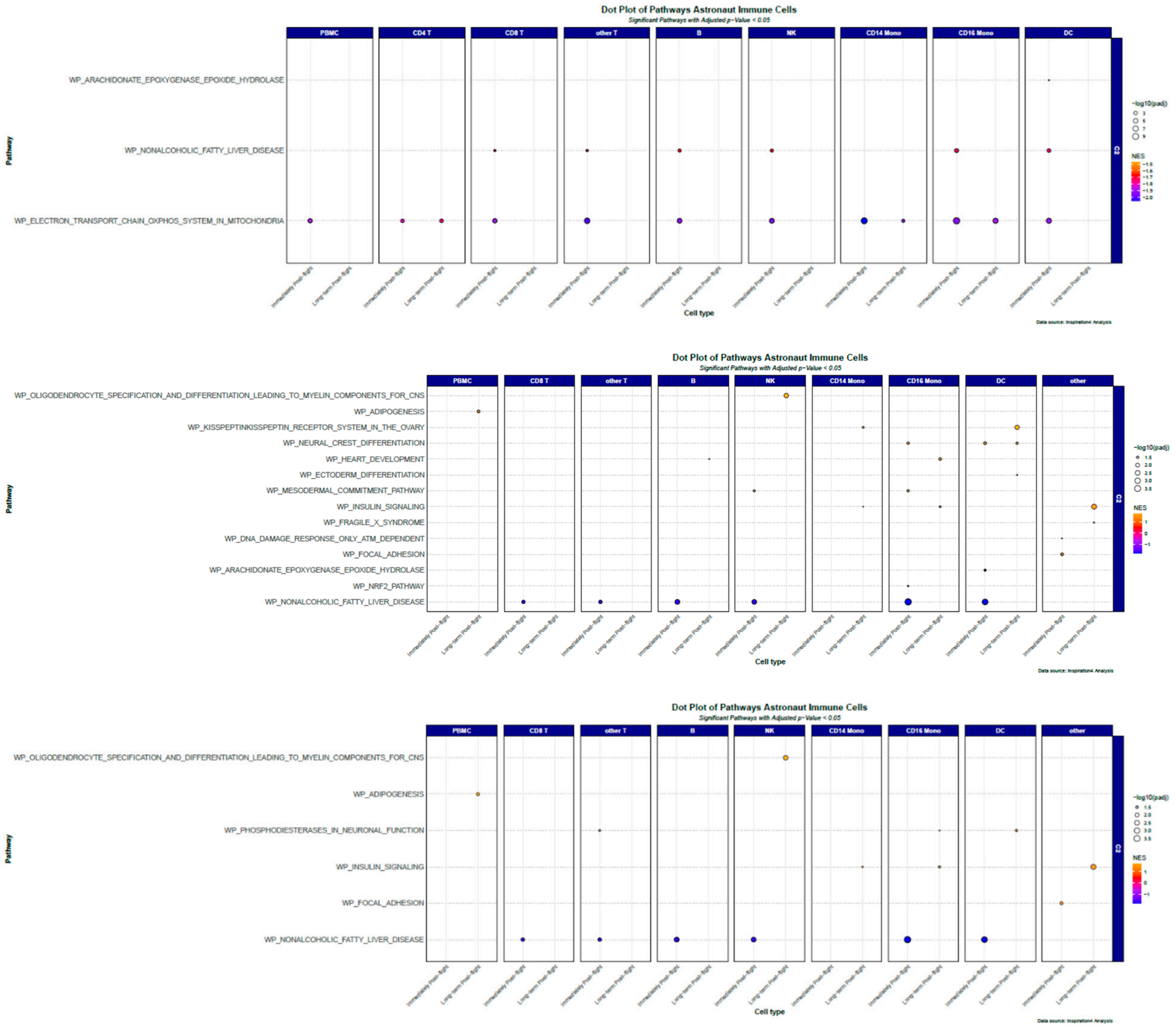

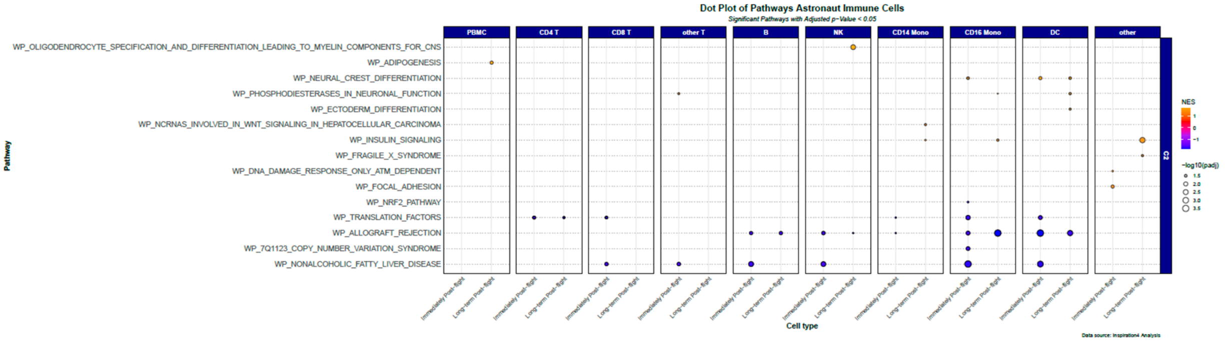
Dot Plot of Pathways Astronaut Immune Cells
Significant Pathways with Adjusted p−Value < 0.05
PBMC
CD4 T
CD8 T
other T
B
NK
CD14 Mono
CD16 Mono
DC
other
C2
Pathway
WP_OLIGODENDROCYTE_SPECIFICATION_AND_DIFFERENTIATION_LEADING_TO_MYELIN_COMPONENTS_FOR_CNS
WP_ADIPOGENESIS
WP_NEURAL_CREST_DIFFERENTIATION
WP_PHOSPHODIESTERASES_IN_NEURONAL_FUNCTION
WP_ECTODERM_DIFFERENTIATION
WP_NCRNAS_INVOLVED_IN_WNT_SIGNALING_IN_HEPATOCELLULAR_CARCINOMA
WP_INSULIN_SIGNALING
WP_FRAGILE_X_SYNDROME
WP_DNA_DAMAGE_RESPONSE_ONLY_ATM_DEPENDENT
WP_FOCAL_ADHESION
WP_NRF2_PATHWAY
WP_TRANSLATION_FACTORS
WP_ALLOGRAFT_REJECTION
WP_7Q1123_COPY_NUMBER_VARIATION_SYNDROME
WP_NONALCOHOLIC_FATTY_LIVER_DISEASE
Immediately Post−flight
Long−term Post−flight
Cell type
NES
1
0
−1
−log10(padj)
1.5
2.0
2.5
3.0
3.5
Data source: Inspiration4 Analysis
